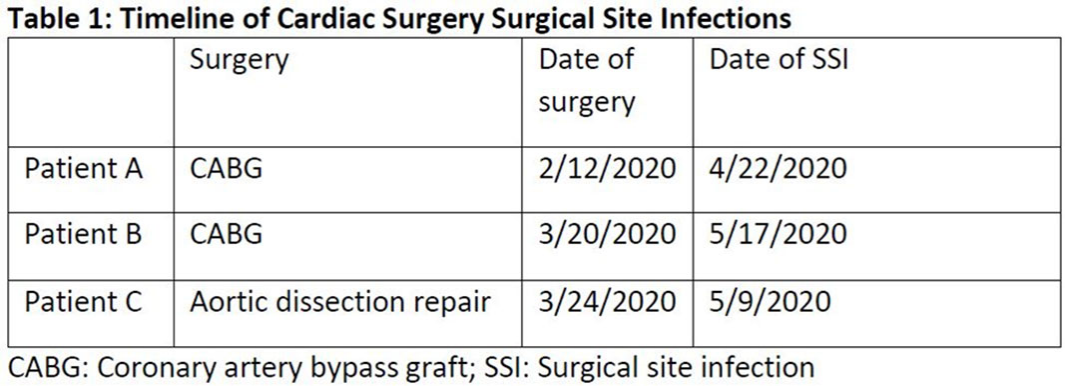# *Mycobacterium abscessus* Surgical Site Infections Due to Modular Cooler-Heater Units in Cardiac Surgery

**DOI:** 10.1017/ash.2021.45

**Published:** 2021-07-29

**Authors:** Ahmed Abdul Azim, Sharon Wright, Bryan Connors, Patrick Gordon, Preeti Mehrotra

## Abstract

**Background:** In the spring of 2020, we identified 3 patients with organ-space surgical site infections (SSIs) secondary to *Mycobacterium abscessus* (Table 1). All 3 patients underwent cardiac surgery in the same operating room (OR) during which the CardioQuip Modular Cooler-Heaters (MCHs) were used. We describe key aspects of our cluster investigation, which ultimately led to release of a national safety alert by the Food and Drug Administration (FDA). **Methods:** For environmental cultures, we obtained samples from 9 MCHs in circulation; 2 scrub sink samples; ice from the OR ice machine; water samples from sinks in the cardiovascular critical care unit, and water samples from floors above the cardiac ORs. All samples were sent for molecular genotyping. For pathway studies, an external environmental engineering team was consulted who conducted smoke pathway tests in 3 different ORs. The team also conducted a particle generator experiment, simulating the set-up of a cardiac bypass surgery case. To assess disinfection practices, we reviewed the manufacturer instructions for use (IFU) protocol of the MCHs and audited our own policies and procedures to ensure compliance. **Results:** For environmental cultures, molecular typing from 5 of 9 MCHs and all 3 patient SSI isolates returned positive for the identical hybrid species *M. abscessus bolleti*. All other samples with mycobacterial growth returned with different species. For pathway studies, the particle-generator experiment demonstrated particle movement from the MCH to the sterile field with facilities-guidelines–compliant OR ventilation and despite MCH manufacturing design. For disinfection practices, despite compliance with the stated IFU, and in consultation with experts, we implemented disinfection of associated Quick-connect devices (otherwise not stated in the IFU), and we also initiated a precleaning step prior to disinfection. **Conclusions:** Our investigation concluded that 3 patients developed SSIs with *Mycobacterium abscessus* that was aerosolized from the CardioQuip MCH. This finding led to the national FDA safety report alerting providers to risks associated with the device and the need for continued vigilance around disinfection. In addition, we implemented other control measures including placement of MCHs outside all ORs; creation of a separate MCH fleet for non-OR use; and use of modified disinfection protocols. To date, no additional cases have been identified.

**Funding:** No

**Disclosures:** None

**Table 1.**